# Human herpesvirus type 1 and type 2 disrupt mitochondrial dynamics in human keratinocytes

**DOI:** 10.1007/s00705-018-3890-y

**Published:** 2018-06-05

**Authors:** Marcin Chodkowski, Izabela Serafińska, Joanna Brzezicka, Anna Golke, Anna Słońska, Małgorzata Krzyżowska, Piotr Orłowski, Piotr Bąska, Marcin W. Bańbura, Joanna Cymerys

**Affiliations:** 1 Division of Microbiology, Department of Preclinical Sciences, Faculty of Veterinary Medicine, Warsaw University of Life Sciences, Ciszewskiego 8, 02-786 Warsaw, Poland; 2 Department of Physiological Sciences, Faculty of Veterinary Medicine, Warsaw University of Life Sciences, Nowoursynowska 159, 02-776 Warsaw, Poland; 3 Military Institute of Hygiene and Epidemiology, Kozielska 4, 01-163 Warsaw, Poland

## Abstract

Mitochondrial movement and distribution throughout the cytoplasm is crucial for maintaining cell homeostasis. Mitochondria are dynamic organelles but can be functionally disrupted during infection. Here, we show that the ubiquitous human pathogens HHV-1 and HHV-2 induce changes in the mitochondrial morphology and distribution in the early and late phases of productive infection in human keratinocytes (HaCaT cells). We observed a decrease in the mitochondrial potential at 2 h postinfection and a decrease in cell vitality at 24 h postinfection. Moreover, we found that mitochondria migrated to the perinuclear area, where HHV-1 and HHV-2 antigens were also observed, mainly in the early stages of infection. Positive results of real-time PCR showed a high level of HHV-1 and HHV-2 DNA in HaCaT cells and culture medium. Our data demonstrate that HHV-1 and HHV-2 cause mitochondrial dysfunction in human keratinocytes.

## Introduction

Human herpesviruses types 1 and 2 (HHV-1 and HHV-2) belong to the subfamily *Alphaherpesvirinae* of the family *Herpesviridae*. Infection with these viruses is widespread in human populations all over the world. To enter its host, a virus must overcome a barrier of mucosal surfaces, skin or cornea. HHV-1 and HHV-2 target keratinocytes during initial entry and establish a primary infection in the epithelium, which is followed by latent infection in neurons. Infections are usually mild but may spread to the central nervous system, causing serious neurological disorders. HHV-1 and HHV-2 have been identified as causative agents of various, mild and even life-threatening diseases, namely, herpes simplex labialis, genital herpes, keratitis and encephalitis [3, 12]. Keratinocytes serve as the first line of defense during skin infections. Their role includes recognition of infectious agents and initiation of innate immune response, which leads to production of cytokines and recruitment of neutrophils. However, viruses have evolved multiple strategies that allow them to escape from the immune response and complete their replication. One of these strategies includes utilization of host cell mitochondria. Mitochondria are organelles that are involved in a variety of metabolic and cellular functions, including Ca^2+^ homeostasis, ATP production, and programmed cell death. They also participate in the synthesis of key metabolites and are the primary source of endogenous reactive oxygen species [6, 16].

Mitochondria form a network distributed throughout the cell [17]. They are dynamic organelles that constantly change their shape, length and movement along cytoskeletal tracks. There are two main processes responsible for mitochondrial homeostasis: fission and fusion. These are crucial for maintenance of a proper number of functional mitochondria. Fusion allows the exchange of DNA, contents and metabolites between neighboring mitochondria. Fission of the mitochondrial network enables the distribution and transport of mitochondria within and outside the cell to a place where energy demand is high. Moreover, fission adjusts apoptosis to eliminate damaged mitochondria [1, 13]. The main proteins responsible for fusion are Mfn-1, Mfn-2 and Opa-1. In mice, knockouts of the genes encoding these proteins results in lethality to embryos and mitochondrial dysfunction. Cells with defective Mfn1 and Mfn2 have a disrupted mitochondrial network and many small, punctate mitochondria. The most important protein participating in the defragmentation of the mitochondrial network in the cell is dynamin-related protein 1 (Drp1), which has GTPase activity. This protein migrates between the cytosol and the mitochondrial network and binds to the mitochondrial outer membrane during fission. Dysregulation of mitochondrial motility has been observed in many human diseases, mainly neurodegenerative disorders, cancer, diabetes, and arrhythmias, and also during ageing [9].

It is believed that mitochondria play a major role in viral infections. Murata et al. [10] have shown that, in Vero cells, mitochondria are recruited to the site of viral replication and morphogenesis. They have also shown that mitochondria migrate to the perinuclear area where HHV-1 tegument was present. It is possible that mitochondria, as energy centers of the cell, provide the energy necessary for replication of the virus. It has also been shown that the mitochondrial potential is stable up to 6 hours postinfection (h p.i.) but decreases during the late phase of infection [10]. In addition, many types of viral proteins have been identified as responsible for the modulation of apoptosis. Alphaherpesviruses, like other large DNA viruses, encode proteins that interfere with mitochondrial function and localization to block the apoptotic pathway. However, little is known about the role of mitochondria in skin cells, which function as the primary barrier and, on the other hand, constitute a very important replication site.

To the best of our knowledge, there are no data available concerning the effects of productive viral infection on mitochondria and the mitochondrial network in keratinocytes, which are crucial in the first steps of herpesviral infection. Therefore, in the present study we investigated the effect of HHV-1 and HHV-2 infection on cell vitality, apoptosis, mitochondrial network rearrangement, and mitochondrial potential in human keratinocytes *in vitro*.

## Materials and methods

### Cell culture and virus strains

Human keratinocytes (HaCaT cells) were cultured as a monolayer using Dulbecco’s modified Eagle’s medium (DMEM; Gibco) supplemented with 10% fetal calf serum (FCS), 2 mM glutamine, 100 U of penicillin per ml and 100 mg of streptomycin per ml, at 37 °C in a humidified atmosphere of 5% CO_2_. Cells were grown to full confluence with a medium change every 2 days. The McIntyre strain of HHV-1 and HHV-2 strain 333 were grown in Vero cell cultures (ATCC no. CRL1587). To produce the virus for the experiments, the Vero cells were infected with HHV-1 or HHV-2 at 0.001 plaque-forming units (PFU)/cell. One hour after infection at 37 °C, the inoculum was removed by aspiration, fresh culture medium was added, and the cells were cultured for 3 days. Culture supernatants were harvested at 72 h after virus challenge, and after three cycles of freezing (-80 °C) and thawing at RT, they were clarified by centrifugation at 800 *g* for 10 min and stored in small volumes at -80 °C. A virus stock suspension containing approximately 10^8^ PFU/ml was used in all the experiments.

HaCaT cells (10^7^ cells per well) were infected with HHV-1 or HHV-2 for 60 min at 37 °C. After adsorption, the inoculum was removed by aspiration and fresh culture medium was added. The cells were then incubated for 2, 24 or 48 hours at 37 °C with 5% CO_2_.

### Real-time cell growth analysis

The growth kinetics, behaviour and morphology of HaCaT cells infected with HHV-1 or HHV-2 were analysed using a JuLI™ Br Live Cell Analyser system for a bright-field image analysis (NanoEnTek, Korea) [4]. HaCaT cells (10^7^ cells per well) were seeded in a 6-well plate and infected with HHV-1 or HHV-2 as described above. Cell-growth images were captured for 48 h at 7-min intervals. Cell confluence analysis was done and a real-time cell growth curve was generated using JuLI Br PC software. All images were captured at objective magnification of ×4.

### Real-time PCR

To determine the number of viral DNA copies per reaction, a standard curve was prepared as described previously [7]. Briefly, fragments of glycoprotein B gene sequence of HHV-1 and HHV-2 were amplified using appropriate primers: HSV-1Fext (GTGATGTTGAGGTCGATGAAGGT) and HSV-1Rext (ACAACGCGACGCACATCAAGGT) and HSV-2Fext (CGTACGATGAGTTTGTGTTGGCGA) and HSV-2Rext (TCAGCTGGTGAGAGTACGCGTA). The products were cloned in pGEM-T Easy Vector. Serial dilutions of recombinant plasmids were prepared ranging from 10 to 10^7^ copies per reaction. Real-time PCR was performed in 96-well plates using a 7500 Real Time PCR System thermocycler (Applied Biosystems) with TaqMan Universal Master Mix II (Applied Biosystems) and probes labelled with JOE as described previously [11].

### Immunofluorescent staining procedures

HaCaT cells seeded on glass coverslips in a 12-well plate were infected with HHV-1 or HHV-2. At 2, 24 and 48 h p.i. (hour postinfection) coverslips were incubated with 100 nM MitoRed (Sigma-Aldrich) for 30 min at 37 °C, then washed three times with culture medium and fixed in 3.7% paraformaldehyde/PBS (Sigma-Aldrich). The presence of viral antigens was detected by means of direct immunofluorescence, using FITC-conjugated polyclonal rabbit anti-herpes simplex virus 1/2 serum (Dako, dilution 1:200).

Additionally, at 2 and 24 h p.i., HaCaT cells were stained for dynamin-related protein 1 (Drp1) detection. At the beginning, cells were washed twice in PBS (Sigma-Aldrich) and fixed in 3.7% paraformaldehyde/PBS (Sigma-Aldrich) for 30 min at room temperature (RT), then permeabilized with 0.5% Triton X-100 (Sigma-Aldrich) solution in PBS. Before staining, fixed HaCaT cells on coverslips were blocked with PBS containing 1% bovine serum albumin (BSA) (Sigma Chemicals) for 30 min at room temperature. The presence of Drp1 was detected by using DNM1L polyclonal antibody (Invitrogen, dilution 1:500) and Alexa Fluor 488 goat anti-rabbit (Invitrogen; dilution 1:250).

Cell nuclei were stained with Bisbenzimidine/Hoechst 33258 according to manufacturer’s recommendations. Afterwards, coverslips were mounted on microscope slides using anti-fade mounting medium (Sigma-Aldrich). Uninfected HaCaT cells served as a negative control. For Drp1 staining, HaCaT cells pre-incubated with Dynasore (GTPase inhibitor; 80 µM/ml) for 60 min before infection served as a positive control. Results were evaluated using a confocal microscope (Leica TCS SP8-WWL).

### Confocal microscopy

Confocal images were acquired using a Leica white light laser scanning confocal microscope (Leica TCS SP8-WWL, KAWA.SKA Sp. z o.o., Poland) with a 63x oil-immersion lens, using excitation at 405 nm, 499 nm, 569 nm for Hoechst, FITC and MitoRed, respectively. Images were captured and converted to 24-bit tiff files for visualization using the Leica Application Suite X (LAS X) software platform (Leica Microsystems).

### Analysis of mitochondrial morphology

For mitochondrial morphology analysis, MiNa Single Image macro was used. This tool allows the number of individuals, number of networks, mean length of branches/rod, mean network size, mean network size per branch, and mitochondrial footprint to be computed. In order to perform this analysis images, obtained by confocal microscopy were used according to the protocol established by Valente et al. [18]. Each analysis was performed on ten cells.

### Image cytometry

Cellular fluorescence was quantified using a NucleoCounter NC-3000 image cytometer (ChemoMetec). The NucleoCounter system was used for evaluation of mitochondrial transmembrane potential (Δψ) and cell vitality at 24 h p.i. In the mitochondrial potential assay, HaCaT cells were stained with JC-1 (cationic dye 5,5,6,6-tetrachloro-1,1,3,3-tetraethylbenzimidazol-carbocyanine iodide; ChemoMetec A/S). First, the suspended HaCaT cells were diluted with PBS to a final concentration of 1.5 ×10^6^ cells/mL. The samples were then incubated with 12.5 mL of a 200 mg/mL solution of JC-1 for 10 min at 37 °C. After incubation, samples were washed twice in PBS and resuspended in 250 mL of a 1 mg/mL solution of 4′,6-diamidino-2-phenylindole in PBS. In the vitality assay (detection of changes in the intracellular level of thiols), HaCaT cells were stained with VitaBright-48 (ChemoMetec A/S), acridine orange (ChemoMetec A/S), and propidium iodide (PI; ChemoMetec A/S). Suspended HaCaT cells were diluted with PBS to a final concentration of 2.0 × 10^9^ cells/mL and were mixed with 5 mL of VitaBright-48·PI·acridine orange. Subsequently, the samples were examined using a Nucleo-Counter NC-3000 according to manufacturer’s instructions. The results were analyzed using the NucleoView NC-3000 software (details of the NucleoCounter NC-3000 design and capabilities are available at www.chemometec.com) [2].

A positive control for mitochondrial potential analysis was prepared by adding CCCP (carbonyl cyanide *m*-chlorophenyl hydrazone; 5 µl per ml of cell culture medium). In parallel, uninfected HaCaT cells served as a negative control.

### Statistical evaluation

The results were statistically evaluated by one-way analysis of variance (ANOVA) using the Student–Newman–Keuls multiple comparisons test and the Tukey–Kramer multiple comparisons test. This analysis was performed using GraphPad Prism^TM^ version 4.03 software (GraphPad Software Inc., San Diego, CA, USA). Statistical differences were interpreted as significant at *P* < 0.05 (*) and highly significant at *P* < 0.01 (**).

## Results

### HHV-1 and HHV-2 replication in HaCaT cells

**Fig. 1:**
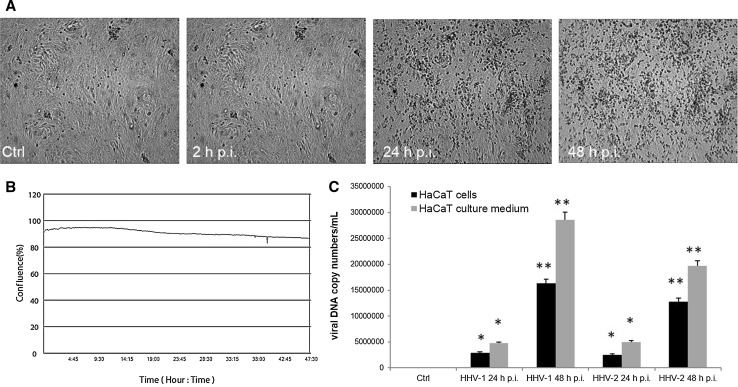
**A and B.** Morphological changes of HaCaT cells infected with HHV-1. Cells were observed for 48 h using a JuLI™ Br Live Cell Analyzer. CPE was manifested as cell destruction and fusion of cells, as confirmed by a growth curve. Objective magnification, x4. **C.** Real-time PCR analysis of viral DNA copy number in HaCaT cells and cell medium during HHV-1 and HHV-2 infection. Statistical differences were interpreted as significant at *P <* 0.05 (*) and *P <* 0.01 (**)

During HHV-1 infection, a cytopathic effect (CPE) was observed as morphology changes in HaCaT cells. After 24 h p.i. and 48 h p.i., we observed a diffuse cytopathic effect, manifesting as cell rounding, shrinking and lysis of individual cells. Interestingly, we did not observe large changes in the confluence of monolayer during the study. At 48 h p.i., the confluence has decreased to around 90% (Fig. 1A and B). Similar results were observed with cells infected with HHV-2 (data not shown).

The quantitative PCR analysis showed a statistically significant increase in the DNA copy number of analyzed viruses in comparison to the uninfected control (Fig. 1C). The highest, statistically significant, increase in the copy number of viral DNA was observed at 48 h p.i. with HHV-1 and HHV-2 (2.8 ± 1.01 × 10^7^ and 1.9 ± 1.31 × 10^7^ copies/ml, respectively; *P* <0.01). We also found a significant increase in the viral DNA copy number in the culture medium at 24 h p.i. and 48 h p.i., which was most probably the result of the release of progeny virions from the cell (Fig. 1C).

### Changes in the mitochondrial network during HHV-1 and HHV-2 infection

**Fig. 2:**
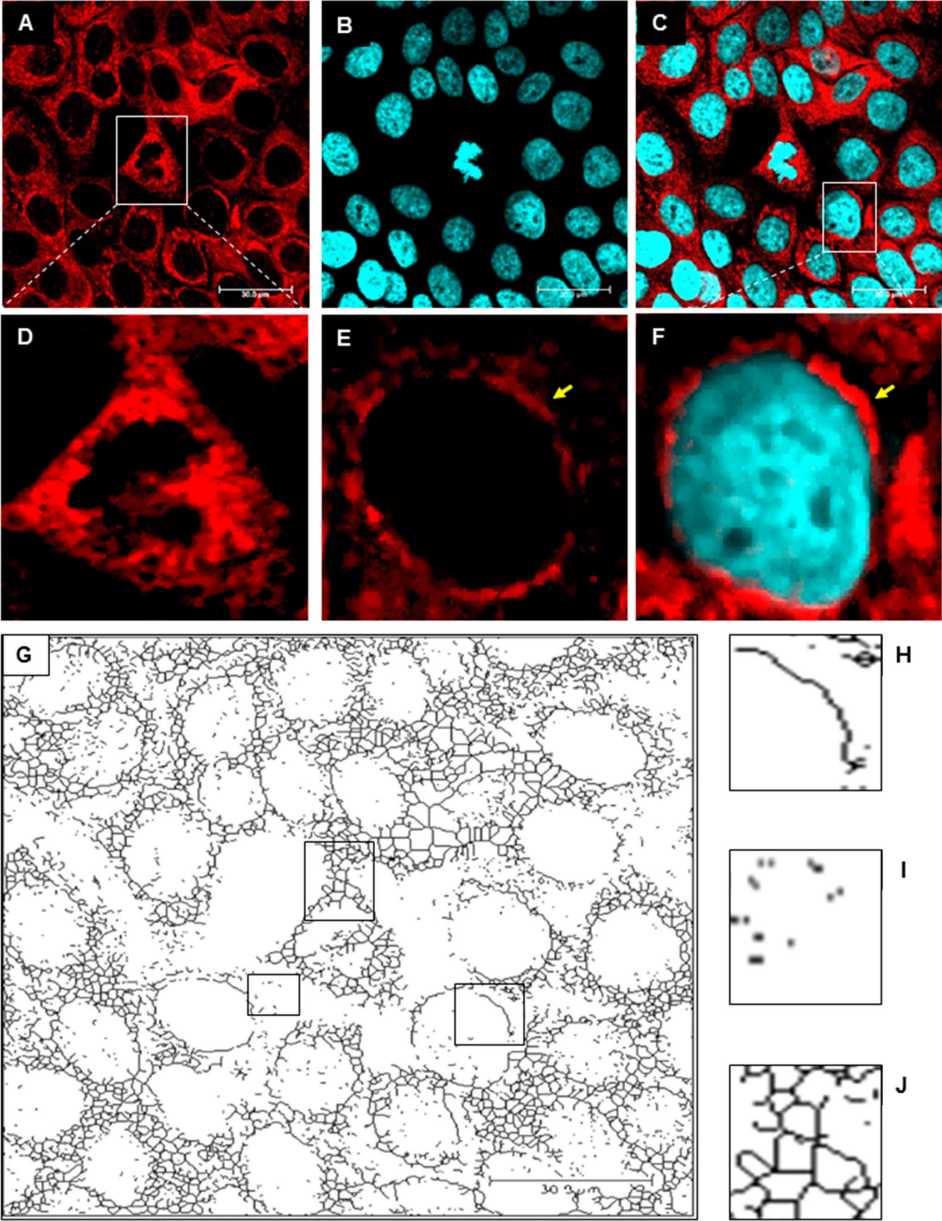
The mitochondrial network in uninfected control HaCaT cells. **A-F.** Immunofluorescence staining of mitochondria (red fluorescence) and the nucleus (blue fluorescence). A yellow arrow indicates tubular mitochondria. **G.** The original image was processed using “unsharp mask”, “CLAHE”, “median”, “binarize” and “skeletonize” for detection different mitochondrial shapes and structures. **H.** Tubular mitochondria. **I.** Punctate mitochondria. **J.** Branched mitochondrial network

In uninfected HaCaT cells, the mitochondrial network was dense, branched and spread evenly throughout the cell (Fig. 2). We observed many tubular, long and highly interconnected mitochondria localized in the subcellular region and a small number of punctate mitochondria. Moreover, we observed fusion of mitochondria of dividing cells, which accumulated in close proximity to the chromosomes (Fig. 2A and D). In uninfected cells, we distinguished three types of mitochondrial shape: tubular (Fig. 2H), punctate (Fig. 2I) and branched mitochondrial network (Fig. 2 J).

**Fig. 3:**
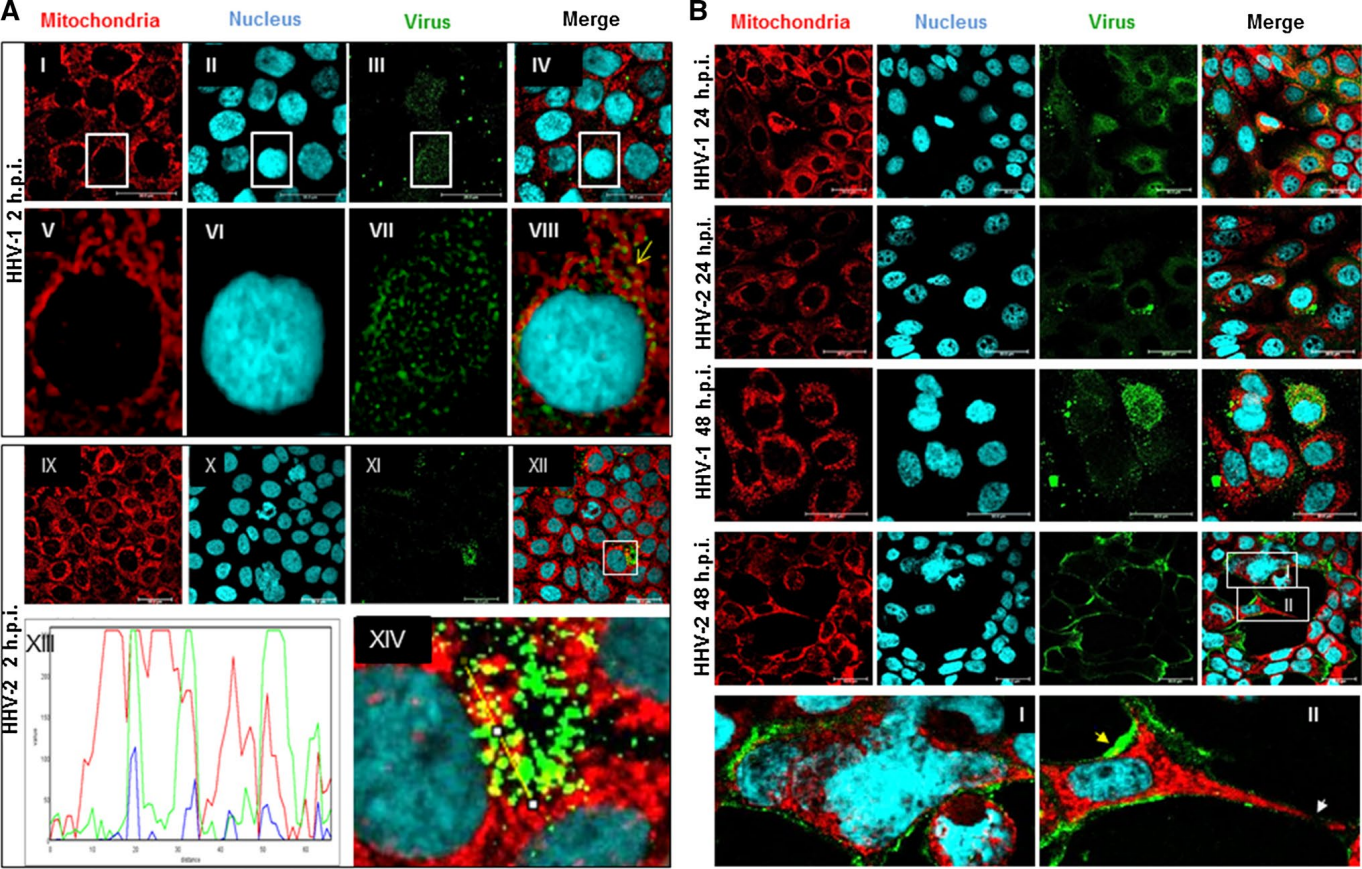
Mitochondrial network organization in HaCaT cells infected with HHV-1 or HHV-2 at 2 (**A**), 24 and 48 h p.i. (**B**). A yellow arrow indicates colocalization of mitochondria with viral antigen (A, VIII). A profile plot of fluorescence signal intensities along the yellow line visible in panel A XIV indicates the colocalization of mitochondria and viral antigen (A, XIII). In panel B (I), the syncytial cytopathic effect in HaCaT cells during infection with HHV-2 at 48 h p.i. is visible. The red arrow in panel B (II) indicates the distribution of viral antigen along the edges of the cell, and the white arrow indicates interruption of the mitochondrial network between infected cells (B, II)

HHV-1 and HHV-2 infection caused changes in the morphology of the mitochondrial network. At 2 h p.i., with both HHV-1 and HHV-2, we observed the interaction of viral particles with the mitochondrial network. The viral antigens were located near the cell nucleus (Fig. 3A), and interestingly, they partially colocalized with mitochondria (Fig. 3A XIII and XIV). At 24 h p.i. with HHV-1 (Fig. 3B), we observed changes in the shape of the mitochondrial network and its distribution within the cell in comparison to uninfected HaCaT cells. The mitochondrial network was organized near the nucleus. At the same time, we observed colocalization of some viral particles with mitochondria. Similar observations were made in the case of infection with HHV-2. After 48 h p.i. we observed an increase in viral replication. During HHV-1 infection, the mitochondrial network was completely fragmented. In the case of HHV-2 infection, after 48 h we observed accumulation of viral antigens within the cells. Moreover, a cytopathic effect in the form of plaques and multinucleated cells was also observed (Fig. 3B I and II).

**Fig. 4:**
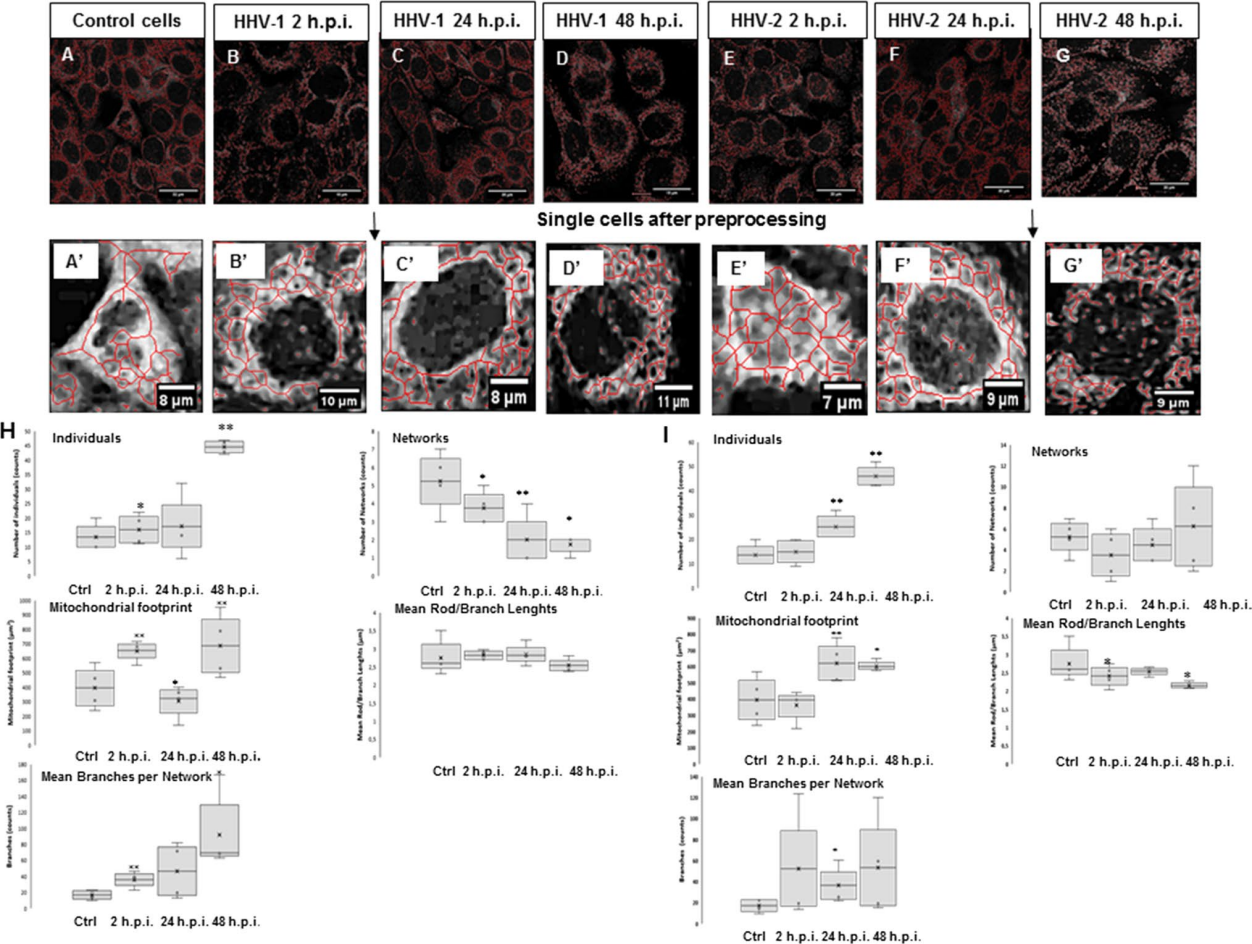
**A-G.** Mitochondrial network analysis performed on 10 HaCaT cells. A’, a single uninfected control cell; B’, a single cell infected with HHV-1 at 2 h p.i; C’, a single cell infected with HHV-1 at 24 h p.i; D’, a single cell infected with HHV-1 at 48 h p.i; E’, a single cell infected with HHV-1 at 2 h p.i.; F’, a single cell infected with HHV-1 at 24 h p.i; G’, a single cell infected with HHV-1 at 48 h p.i. **H and I.** Summary statistics for all infected and control HaCaT cells. The box plot shows median (horizontal lines), first-to-third quartile (box), and extreme values (%) (**, *P* < 0.01; *, *P* < 0.05). Each analysis was performed on 10 cells

Examples of cells selected for mitochondrial morphology analysis are shown in Fig. 4A-G’. At 2 h p.i., with HHV-1, we observed a decrease in the number of mitochondrial networks, together with an increase in the number of individual mitochondrial objects. In addition, both the percentage of cross-linked mitochondria and the length of the network branches decreased. Moreover, the total area of mitochondria decreased. At 24 h p.i., the number of mitochondrial objects increased, and we observed a decrease in the number of mitochondrial networks. We also observed a reduction in the total mitochondrial area. At 48 h p.i., we observed a significant increase in the number of mitochondrial objects. The mean number of branches per network was reduced at 2, 24, and 48 h p.i., but the results were not statistically significant. HHV-1 infection caused various changes in the morphology of the mitochondrial network in HaCaT cells. This analysis shows that the mitochondrial network underwent fission and was fragmented after infection (Fig 4H).

HHV-2 infection caused changes similar to those caused by HHV-1. We observed a gradual increase in the number of mitochondria, which reached a maximum at 48 h p.i. We also observed changes in mitochondrial cross-linking, as well as a decrease in the length of the mitochondrial branches, but these differences were statistically insignificant. At 24 and 48 h p.i., a statistically significant decrease in the overall mitochondrial surface area was observed (Fig. 4I).

**Fig. 5:**
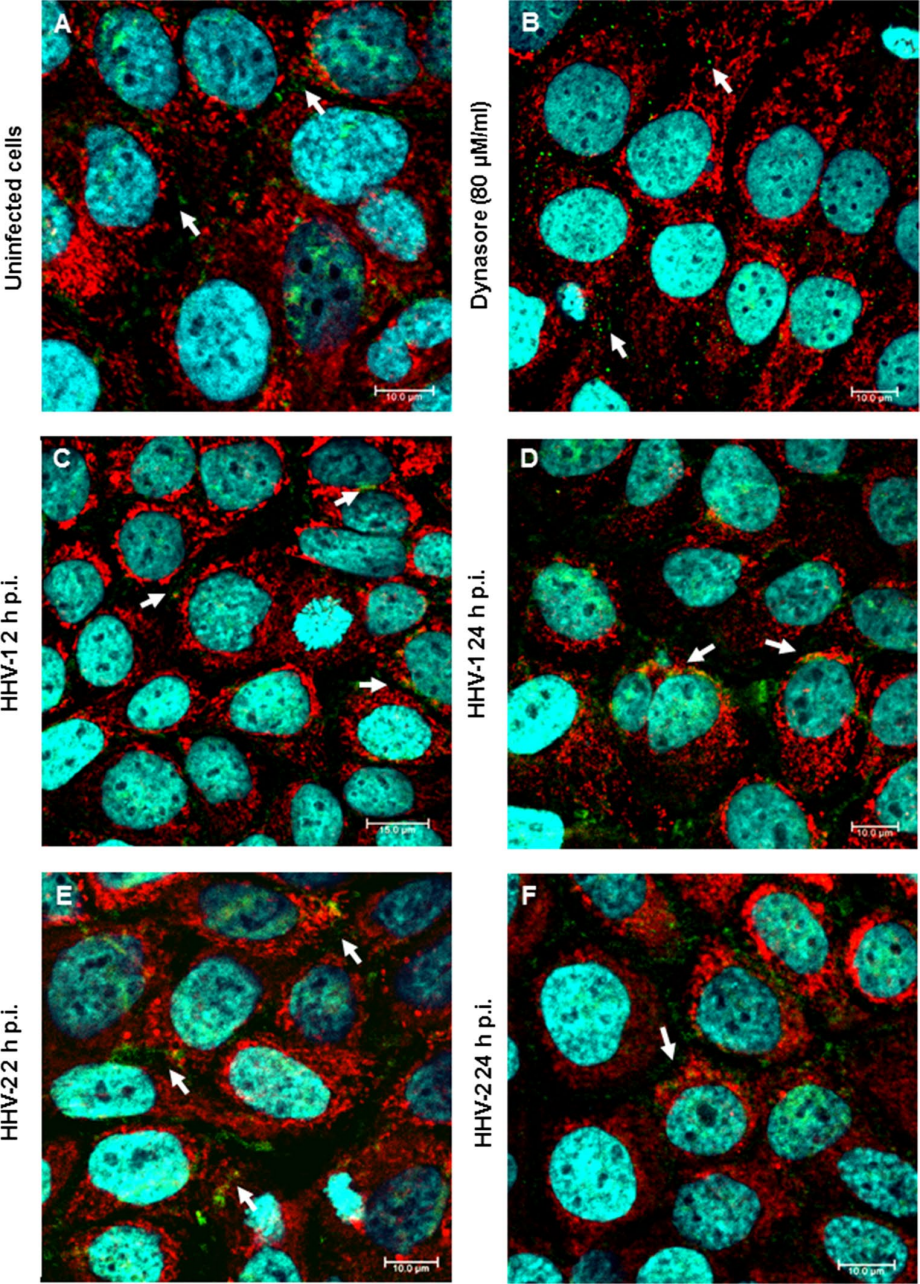
Localization of Drp1 in HaCaT cells. Immunofluorescence staining was used to examine mitochondrial translocation of Drp1 fission protein (white arrows indicate localization of Drp1). **A**. Uninfected HaCaT cells. **B.** Cells treated with Dynasore. **C and D.** Cells infected with HHV-1. **E and F.** Cells infected with HHV-2. Drp1, green fluorescence; mitochondria, red fluorescence; nuclei, blue fluorescence. Objective magnification, x63

We then investigated the distribution of Drp1 in HaCaT cells. In uninfected control cells, Drp1, as well as the mitochondrial network, was distributed evenly in the cytoplasm (Fig. 5A). After 24 h of treatment with Dynasore, which is an inhibitor of GTPases, we observed a decrease in the Drp1 level in treated cells and the presence of tubular mitochondria (Fig. 5B). During infection, we observed that Drp1 was partially translocated from the cytoplasm to the outer membrane of the mitochondria. After 2 h p.i., with both HHV-1 and HHV-2, we observed a colocalization of Drp1 with mitochondria localized in perinuclear area. After 24 h p.i. we observed a decrease in Drp1 protein expression in infected cells together with progressive disintegration of the mitochondrial network (Fig. 5C-F).

### The mitochondrial potential and vitality of HaCaT cells during HHV-1 and HHV-2 infection

**Fig. 6:**
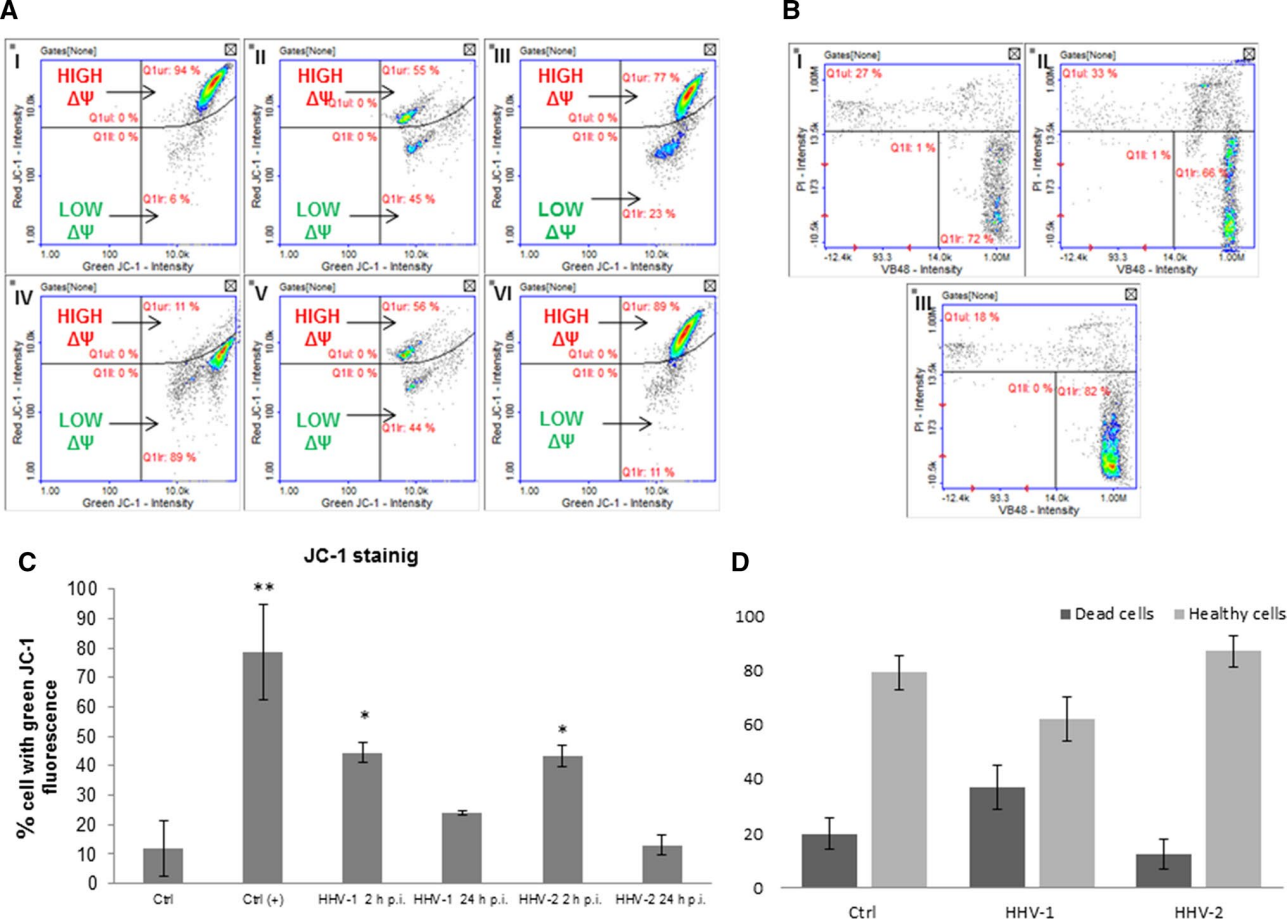
**A and C.** Mitochondrial membrane potential (ΔΨ) of HaCaT cells. Uninfected control cells have a high mitochondrial potential (I). CCCP-treated HaCaT cells (positive control) have a low mitochondrial potential (IV). The level of green fluorescence in cells is indicated as a percentage (**, *P* < 0.01; *, *P <* 0.05). II, HaCaT cells infected with HHV-1 at 2 h p.i.; III, HaCaT cells infected with HHV-1 at 24 h p.i.; V, HaCaT cells infected with HHV-2 at 2 h p.i.; VI, HaCaT cells infected with HHV-2 at 24 h p.i. **B and D.** Cell vitality assay. HaCaT cells were stained with VitaBright -48 ™ (VB-48™), acridine orange (AO), and propidium iodide (PI). I, control cells; II, HaCaT cells infected with HHV-1 at 24 h p.i. III, HaCaT cells infected with HHV-2 at 24 h p.i. **D.** Level of dead and live cells

In this assay, changes in the mitochondrial potential that occurred during HHV-1 or HHV-2 infection were evaluated (Fig. 6A, C). Considering the pivotal role of mitochondria in orchestrating the apoptotic pathway, we measured the mitochondrial potential using the JC-1 method. The principle of the test depends on the fact that JC-1, a mitochondrial-potential-sensitive dye, accumulates in the matrix of mitochondria by forming J-aggregates with red fluorescence when the mitochondrial potential is high and becomes a monomer with green fluorescence when it is low. HaCaT cells treated with CCCP served as a positive control (Fig. 6A IV). In these cells, a low mitochondrial potential was observed. Cells treated with CCCP exhibited the characteristics of cells with dysfunctional mitochondria; JC-1 was present in the cytosol in its monomeric form, emitting green fluorescence. During infection with both HHV-1 and HHV-2 (2 and 24 h p.i.), we observed changes in mitochondrial potential. The most significant decrease was observed in HaCaT cells at 2 h p.i. However, at 24 h p.i., a decrease in the mitochondrial potential had also occurred, but it was not statistically significant and was similar to the values obtained with the negative control (Fig. 6A, C). It was also found that HHV-1 and HHV-2 infection caused a reduction in the vitality of HaCaT cells at 24 h p.i. (Fig. 6B and D). The decrease in vitality was more pronounced in the case of HHV-1 infection than after infection with HHV-2.

## Discussion

The major target cells during primary and recurrent HHV-1/HHV-2 infections are cells of epithelial and neuronal origin. During the initial exposure, HHV-1 and HHV-2 use mucosal epithelial cells, including epidermal keratinocytes, as the primary portal of entry, and infection then spreads through the epithelium. Additionally, after episodic reactivation from latency established in neurons, newly replicating viruses infect epithelial cells, often leading to recurrent herpetic lesions. The productive cycle of HHV-1 and HHV-2, confirmed by qPCR analysis, caused morphological changes in infected cells (Fig. 1). These changes were manifested as a loss of intercellular connections between HaCaT cells and the presence of syncytial cells at 48 h p.i., which was also observed in previous studies [12]. Overall, the cytopathic effect caused by HHV-1 and HHV-2 in the late stages of infection was very similar.

In this study, we performed an analysis of mitochondrial dynamics during HHV-1 and HHV-2 infection in human keratinocytes. Changes in the mitochondrial network were apparent in the early stage of infection with HHV-1 or HHV-2. We also observed the migration of mitochondria to the perinuclear area, both in the early and late stages of infection. Similar results were obtained previously, and it was also shown that microtubules are responsible for the transport of mitochondria. Moreover, the migration of mitochondria to the perinuclear area during infection with HHV-1 was inhibited after addition of the microtubule inhibitor nocodazole [10]. These results have provided new insights into the various strategies of cytoskeleton utilization that are used during herpesviral infections. Apparently, the cytoskeleton is not only necessary for transport of the virions to the nucleus, as has been described before, but also supports spatial rearrangement of cellular organelles in order to facilitate the replication cycle [5, 15].

After HHV-1 or HHV-2 infection, we also observed changes in mitochondrial morphology. In control cells, the mitochondria were elongated and branched. Murata et al. also observed the changes in the mitochondrial area, but they did not characterize these changes [10]. According to ImageJ analysis, mitochondria were significantly affected by HHV-1 or HHV-2 infection. Primarily, we observed defragmentation of the mitochondrial network and an increased number of punctate mitochondria. We also detected colocalization of punctate mitochondria with viral antigen. In the case of HHV-1, in the late stage of infection, there was a decrease in the number of mitochondrial networks, but we did not observe a significant change in the case of HHV-2 infection. These changes were not observed in cells in which viral antigen was not detected. Similar changes, including an increase in the number of punctate mitochondria, were also observed previously in the case of infection with Human herpesvirus type 5 (HHV-5) [8]. Our results suggest that HHV infection causes increased fission of the mitochondrial network. Moreover, we observed a translocation of Drp1 to the perinuclear area and binding of this protein to mitochondrial outer membrane. This may indicate that viral infection increases the activity of this protein, stimulating the defragmentation of mitochondrial network.

It is believed that one of the early indicators of apoptosis is the permeabilization of the mitochondrial membrane. For that reason, we evaluated the mitochondrial potential using JC-1 dye. We believe that the internal mitochondrial potential decreased as a consequence of changes in the mitochondrial network. It was the result of rearrangements of the network as well as changes in the distribution of mitochondria in the early stages of infection. Interestingly, the mitochondrial potential had been stabilized by 24 h p.i. At 2 h p.i., we observed a large loss in the mitochondrial potential after infection with both HHV-1 and HHV-2. These results showed that the most significant changes in the mitochondrial network occurred in the early stages of infection. We did not observe a substantial decrease in the mitochondrial potential at 24 h p.i. Similar results were obtained by Kramer and Enquist in the case of Suid herpesvirus 1 (SuHV-1) infection, which suggests that infection with alphaherpesviruses may not cause permeabilization of mitochondrial membranes [6].

In addition, we measured the level of free thiols, which changes during apoptosis or other pathological processes. The assay provides a reliable and fast way to evaluate cell viability, as an appearance of free thiols is the early indicator of apoptosis [14]. In our study, we did not observe a significant change in the level of free thiols after infection with either HHV-1 or HHV-2. This may indicate that only a small percentage of infected HaCaT cells undergo apoptosis.

In conclusion, our results suggest that productive HHV-1 and HHV-2 infections cause changes in the mitochondrial network in comparison to uninfected control cells. These changes manifest themselves mainly as defragmentation of the mitochondrial network, an increase in the number of punctate mitochondria, and a decrease in the number of tubular mitochondria. In addition, in the early stage of infection, mitochondrial membrane permeabilization occurs, which may be associated with rearrangement of the network. These results indicate that mitochondria are utilized in the early stages of HHV-1 and HHV-2 replication, as evidenced by the increased transport of punctate mitochondria to the replication site and colocalization of mitochondria with viral antigens in the perinuclear area. It is, however, worth stressing that viral infection does not destroy the mitochondrial network completely, which ensures survival of the cell and enables viruses to complete their replication cycle.
